# Role of CXCL16 in BLM-induced epithelial–mesenchymal transition in human A549 cells

**DOI:** 10.1186/s12931-021-01646-7

**Published:** 2021-02-06

**Authors:** Zhenzhen Ma, Chunyan Ma, Qingfeng Zhang, Yang Bai, Kun Mu, Xiangyuan Liu, Qingrui Yang

**Affiliations:** 1 Department of Rheumatology and Immunology, Shandong Provincial Hospital, Cheeloo College of Medicine, Shandong University, Jinan, 250021 Shandong China; 2 Department of Rheumatology and Immunology, Shandong Provincial Hospital Affiliated to Shandong First Medical University, Jinan, 250021 Shandong China; 3 Department of Rheumatology and Immunology, Peking University Third Hospital, Beijing, 100191 China; 4 Department of Central Laboratory, Shandong Provincial Hospital Affiliated to Shandong First Medical University, Jinan, 250021 Shandong China; 5 Department of Pathology, Qilu Hospital, Shandong University, Jinan, 250012 China

**Keywords:** Pulmonary fibrosis, Chemokines, CXCL16, Epithelial–mesenchymal transition

## Abstract

Alveolar epithelial cells play an essential role in the initiation and progression of pulmonary fibrosis, and the occurrence of epithelial–mesenchymal transition (EMT) may be the early events of pulmonary fibrosis. Recent studies have shown chemokines are involved in the complex process of EMT, and CXC chemokine ligand 16 (CXCL16) is also associated with many fibrosis-related diseases. However, whether CXCL16 is dysregulated in alveolar epithelial cells and the role of CXCL16 in modulating EMT in pulmonary fibrosis has not been reported. In this study, we found that CXCL16 and its receptor C-X-C motif chemokine receptor 6 (CXCR6) were upregulated in bleomycin induced EMT in human alveolar type II-like epithelial A549 cells. Synergistic effect of CXCL16 and bleomycin in promoting EMT occurrence, extracellular matrix (ECM) excretion, as well as the pro-inflammatory and pro-fibrotic cytokines productions in A549 cells were observed, and those biological functions were impaired by CXCL16 siRNA. We further confirmed that CXCL16 regulated EMT in A549 cells via the TGF-β1/Smad3 pathways. These results indicated that CXCL16 could promote pulmonary fibrosis by promoting the process of EMT via the TGF-β1/Smad3 signaling pathway.

## Background

Pulmonary fibrosis is a progressive and destructive lung disease with various causes, which seriously endangers patients’ health and life. It is now well-recognized that "pulmonary fibrosis" covers a broad range of lung diseases, including most topically the idiopathic pulmonary fibrosis (IPF), and connective tissue disease-associated interstitial lung disease (CTD-ILD) [1]. With high morbidity and mortality, the median survival time of IPF after diagnosis was about 2–3 years in the absence of efficient treatment [2]. Combined ILD is an important predictor of outcomes in patients with CTD, and it is also one of the most common causes of death in patients with CTD [3, 4]. Due to the lack of effective treatment to improve its adverse outcome, it is urgent to deepen the study of the mechanism of pulmonary fibrosis and develop new treatment strategies on this basis.

The pathogenesis and pathological process of pulmonary fibrosis is always divided into four parts: injury of the lung tissue with various causes, release of diverse pro-inflammatory and pro-fibrotic mediators, destruction of the tissue structures and the subsequent tissue repairs. However, repeated and abnormal repair of the lung tissue results in the disorder of the internal environment and excessive deposition of extracellular matrix (ECM), and finally leads to the occurrence of pulmonary fibrosis [5]. Some studies have shown that alveolar macrophages, alveolar epithelial cells and pulmonary interstitial cells all participate in the development of pulmonary fibrosis by secreting inflammatory cytokines and mediators, directly or indirectly [6]. Among them, alveolar epithelial cells play a key role in the initiation and progression of pulmonary fibrosis, and it may undergo EMT to contribute to foci development in the early in the fibrotic disease [7]. Myofibroblasts (MFB) are regarded as the main perpetrators of fibrosis as they appear to be the major source of ECM proteins, and EMT is an important source of myofibroblasts in fibrosis related diseases [8]. In vivo lineage-tracing studies have showed that about 1/3 of MFB originated from epithelial cells in the pulmonary fibrosis models prepared by single bleomycin (BLM) injection [9], while about 1/2 of MFB originated from epithelial cells in the models prepared by multiple BLM injection [10]. Hence, to explore the underlying mechanisms of EMT in pulmonary fibrosis may provide an important basis for the treatment of pulmonary fibrosis and related diseases.

In the past few decades, some new understandings and important advances have been made in the study of the molecular mechanisms of EMT, especially the role of chemokines in the complex EMT process. For example, the CXC chemokine 7 (CXCL7) inhibits growth and migration of oral tongue squamous cell carcinoma cells via the EMT signaling pathway [11], CC-Chemokine Ligand 18 (CCL18) induces EMT in lung cancer and elevates the invasive potential [12], and CXC chemokine 9 (CXCL9) regulates TGF-β1-induced EMT in human alveolar epithelial cells [13]. CXCR6, the only receptor for CXCL16, predicts poor prognosis and promotes cancer metastasis in gastric cancer through EMT induction [14]. Specifically, we found that CXCL16/CXCR6 in the lung tissues of Saline-treated wild-type mice was only expressed in a small amount in the airway epithelial cells and the vascular smooth muscle cells, while CXCL16/CXCR6 in the lung tissues of BLM-treated wild-type mice was widely expressed in the airway and alveoli epithelium cells and the lung interstitium, accompanied by the infiltration of CXCR6 positive lymphocytes in the alveolar cavity (Additional file 1: Fig. S1). However, whether CXCL16 is also dysregulated in the human alveolar epithelial cells and the role of CXCL16 in modulating EMT in pulmonary fibrosis has not been reported. In this study, the potential role of CXCL16 in regulating EMT in BLM-induced pulmonary fibrosis in human A549 cells was investigated.

## Materials and methods

### Cell culture

Human alveolar type II-like epithelial A549 cells were purchased from the National Infrastructure of Cell Line Resource (Beijing, China). A549 cells were maintained in Dulbecco Modified Eagle Medium (DMEM) containing 10% fetal bovine serum (FBS), and were cultured at 37 °C in a humidified incubator with 5% CO_2_.

### Cell viability assay

A549 cells (5 × 10^3^/well) were seeded in 96-well plates for 4 h and then incubated with different concentrations of BLM (Selleck Chemicals, USA) or CXCL16 (PeproTech, USA) for 24 h. The effect of BLM or CXCL16 on cell viability was detected by using the CellTiter 96^®^AQueousOne Solution Cell Proliferation Assay (MTS assay, USA) according to the manufacturer’s protocol.

### Real-time PCR

Total RNA was extracted by using the TRIzol reagent (Invitrogen, USA) according to the manufacturer’s protocol, and 1 μg of total RNA was reverse-transcribed into cDNA by using the FastQuant RT Kit (Tiangen, China). RT-PCR was carried out by using the QuantStudio™ 5 Real-Time PCR System (Thermo Fisher Scientific, USA) with Talent qPCR PreMix (Tiangen, China). For each experiment, at least three parallel measurements were carried out, and GAPDH served as a loading control. The specific primers used in this study were shown in Additional file 1: Table S1.

### Western blot

Total protein was harvested by using the RIPA buffer and was determined with a BCA protein assay kit. The protein samples were separated (40 μg) via 10% SDS-PAGE and transferred to PVDF membranes (Millipore, USA). After blocking with 5% non-fat milk for 2 h, the membranes were incubated with the relevant antibody for 12–14 h at 4 °C and were incubated with the specific secondary antibodies for 1 h at room temperature. The membranes were scanned using an Odyssey Sa Imaging System (LI-COR Biosciences, USA). GAPDH was used as loading control. The specific antibodies used in this study were shown in Additional file 1: Table S2.

### Immunofluorescence

A549 cells (5 × 10^3^/well) were seeded in confocal dishes (NEST, China) and were treated with BLM for 24 h. After washed with PBS, the cells were fixed with 4% paraformaldehyde for 30 min, and blocked with 2% goat serum for 1 h at room temperature. The cells were incubated overnight at 4 °C with specific antibodies (CXCL16, 1:100; CXCR6, 1:100), and then incubated with 488-labeled goat anti-rabbit IgG for 1 h at room temperature. DAPI (SIGMA) was counter-stained and images were acquired on a laser confocal microscope (Leica TCS SP8, Germany).

### Enzyme-linked immunosorbent assay (ELISA)

Levels of interleukin-6 (IL-6), interleukin-8 (IL-8), interleukin-1β (IL-1β) and the tumor necrosis factor-alpha (TNF-α) in cell culture supernatant were separately detected by the Human High Sensibility ELISA Kit (MultiSciences, China). Soluble CXCL16 (sCXCL16) and the transforming growth factor-β1 (TGF-β1) in cell culture supernatant were separately detected by the Human CXCL16/TGF-β1 Immunoassay Quantikine ELISA kit (R&D Systems, USA) according to the manufacturer’s protocol.

### Transient transfection

CXCL16 siRNA (Santa Cruz, USA), Scramble control and positive control (Ribobio, China) were transfected into A549 cells by using Lipofectamine RNAiMAX (Invitrogen, USA) following the manufacturer's instructions, and the transfection efficiency was detected by western blot 48 h later.

### Statistical analysis

Statistical analyses were performed with SPSS (version 22.0) and Graphad Prism (version 7.0). All experiments were repeated at least three times. Data was expressed as mean ± standard deviation (SD). Analysis of variance (ANOVA) with post hoc Dunnett’s correction was performed to compare continuous variables. The Mann–Whitney U-test was used for non-parametric comparisons. P < 0.05 was considered statistical significant.

## Results

### BLM-treated A549 cells have undergone EMT

**Fig. 1:**
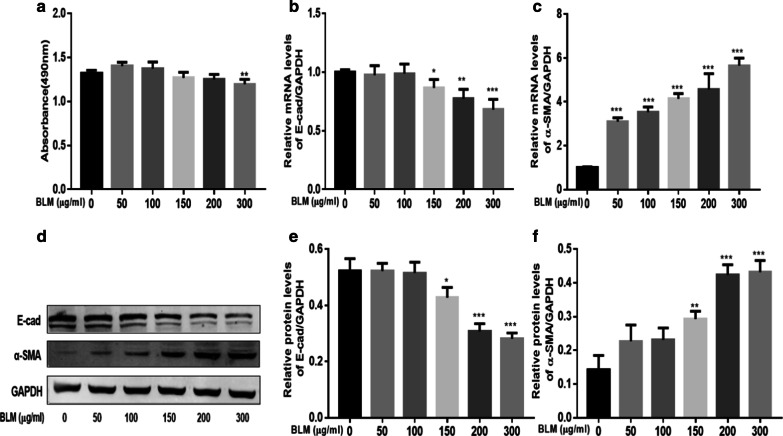
BLM-treated A549 cells have undergone EMT. A549 cells were incubated with different concentrations of BLM for 24 h and the MTS assay was used to detect cell viability (**a**). Levels of EMT-related markers were detected, the mRNA levels of E-cad (**b**) and α-SMA (**c**) were detected by real-time PCR, the protein levels of E-cad (**e**) and α-SMA (**f**) were detected by western blot (**d**). *EMT* epithelial–mesenchymal transition, *BLM* Bleomycin, *E-cad* E-cadherin, *α-SMA* α-smooth muscle actin; GAPDH served as a loading control; *P < 0.05, **P < 0.01, ***P < 0.001. Significantly different versus 0 μg/ml. Data represented three independent experiments

Results from the MTS assay indicated that BLM inhibited the proliferation of A549 cells in a dose-dependent manner and the appropriate concentrations of BLM in treating A549 cells for 24 h were 200 μg/ml (Fig. 1a). Next, phenotypic markers associated with EMT including α-SMA and E-cadherin was detected by PCR and western blot. Results showed that with the increase of the BLM concentrations, increased levels of α-SMA expression and decreased levels of E-cadherin expression have been observed, both in mRNA and protein levels (Fig. 1b–f). These data confirmed that BLM -treated human A549 cells have undergone EMT.

### CXCL16 and its receptor CXCR6 were upregulated in BLM-induced EMT in human A549 cells

**Fig. 2:**
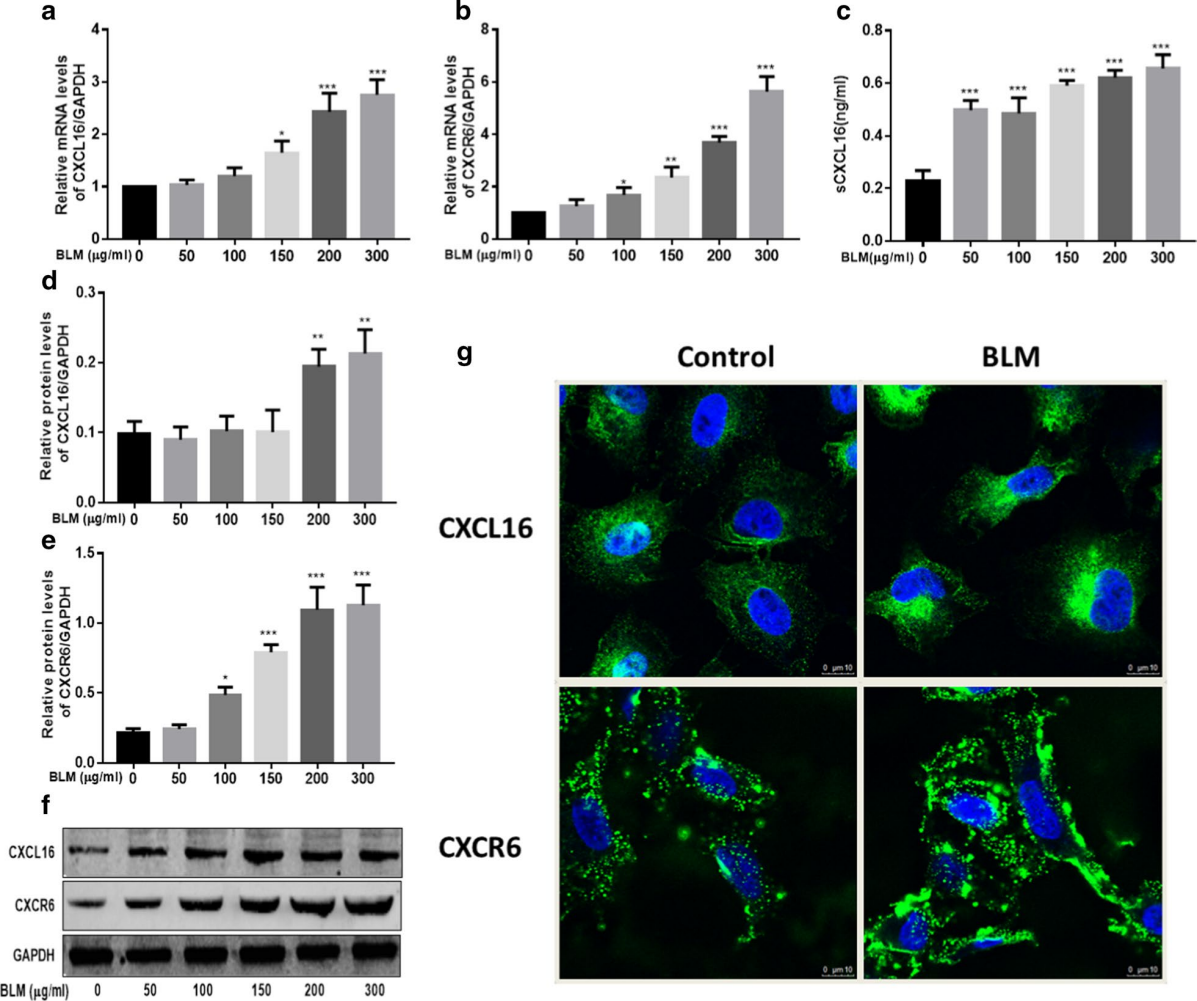
CXCL16 and its receptor CXCR6 were upregulated in BLM-induced EMT in A549 cells. A549 cells were treated with different concentrations of BLM for 24 h. CXCL16 (**a**) and CXCR6 (**b**) mRNA levels were detected by RT-PCR, the expression of sCXCL16 in the cell culture supernatant was detected by ELISA (**c**), and protein levels of CXCL16 (**d**) and CXCR6 (**e**) were detected by western blot (**f**). Expression of CXCL16 and CXCR6 were also detected by cellular immunofluorescence (**g**). *BLM* Bleomycin; GAPDH served as a loading control. *P < 0.05, **P < 0.01, ***P < 0.001. Significantly different versus 0 μg/ml. Data represented three independent experiments. Scale bar, 100 μm

Next, we detected the expression of CXCL16 and its receptor CXCR6 in BLM-induced EMT in A549 cells, by RT-PCR, western blot and immunofluorescence. We found that mRNA levels of CXCL16 and CXCR6 were markedly increased in A549 cells when treated with BLM, with similar results obtained in western blot and immunofluorescence (Fig. 2). The results indicated that CXCL16 and its receptor CXCR6 were up-regulated in A549 cells and might participate in the development of BLM-induced EMT in human A549 cells.

### Synergistic effect of CXCL16 and BLM on EMT occurrence, ECM excretion, pro-inflammatory and pro-fibrotic cytokines productions in human A549 cells

**Fig. 3:**
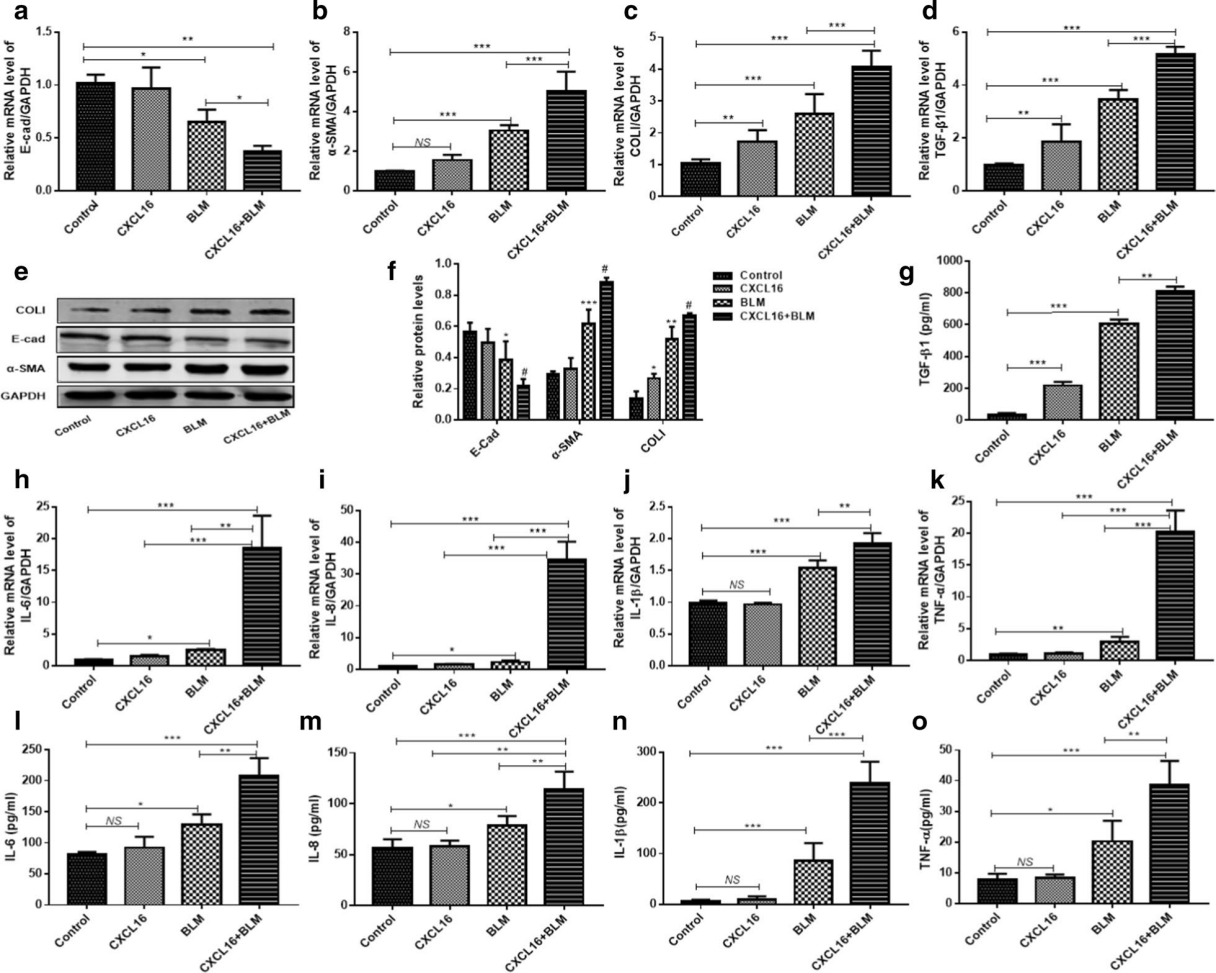
After co-stimulation of CXCL16 (200 ng/ml) and BLM (200 μg/ml) in A549 cells for 24 h, the mRNA levels of E-cad (**a**), α-SMA (**b**),COLI (**c**), TGF-β1 (**d**), IL-6 (**h**), IL-8 (**i**), IL-1β (**j**) and TNF-α (**k**) were determined by RT-PCR and the protein levels of E-cad, α-SMA and COLI were determined by western blot (**e**, **f**).The levels of TGF-β1 (**g**), IL-6 (**l**), IL-8 (**m**), IL-1β (**n**) and TNF-α (**o**) in the supernatants of A549 cells were detected using the corresponding ELISA kit. *BLM* Bleomycin, *E-cad* E-cadherin, *α-SMA* α-smooth muscle actin, *TGF-β1* the transforming growth factor-β1, *COLI* collagen I, *IL-6* interleukin-6, *IL-8* interleukin-8, *IL-1β* interleukin-1β, *TNF-α* the tumor necrosis factor-alpha. GAPDH served as a loading control. *P < 0.05, **P < 0.01, ***P < 0.001, ^#^P < 0.05; *NS* not significant. *Significantly different versus the control group. ^#^Significantly different versus the BLM group. Data represented three independent experiments

To further examine whether CXCL16 has a synergistic effect with BLM on the EMT occurrence and ECM excretion in human A549 cells, the cells were co-stimulated with CXCL16 and BLM. First, the appropriate concentrations of CXCL16 in treating A549 cells for 24 h were 200 ng/ml, as confirmed by the MTS assay. Co-stimulation of 200 ng/ml CXCL16 and 200 μg/ml BLM was implemented for 24 h in A549 cells. Results showed that the up-expression of α-SMA and the down-expression of E-cadherin was observed in the co-stimulation group, compared with single stimulation with BLM, which indicated that CXCL16 and BLM synergistically promoted the occurrence of EMT (Fig. 3a, b). Through the processes of EMT, epithelial cells often lose their own properties and present as a mesenchymal phenotype with the ability to secrete collagen. Thus, the expression of collagen I (COLI) was detected, and the expression of COLI in both mRNA and protein levels were found to be elevated (Fig. 3c, e). We also found that IL-6, IL-8, IL-1β, TNF-α and TGF-β1 mRNA and protein levels were markedly increased in A549 cells in the co-stimulation group, compared with the BLM group (Fig. 3h–o). The above data indicated that CXCL16 had a synergistic effect with BLM in promoting EMT occurrence, ECM excretion, pro-inflammatory and pro-fibrotic cytokines productions in A549 cells.

### CXCL16 regulated BLM-induced EMT and ECM excretion in human A549 cells

**Fig. 4:**
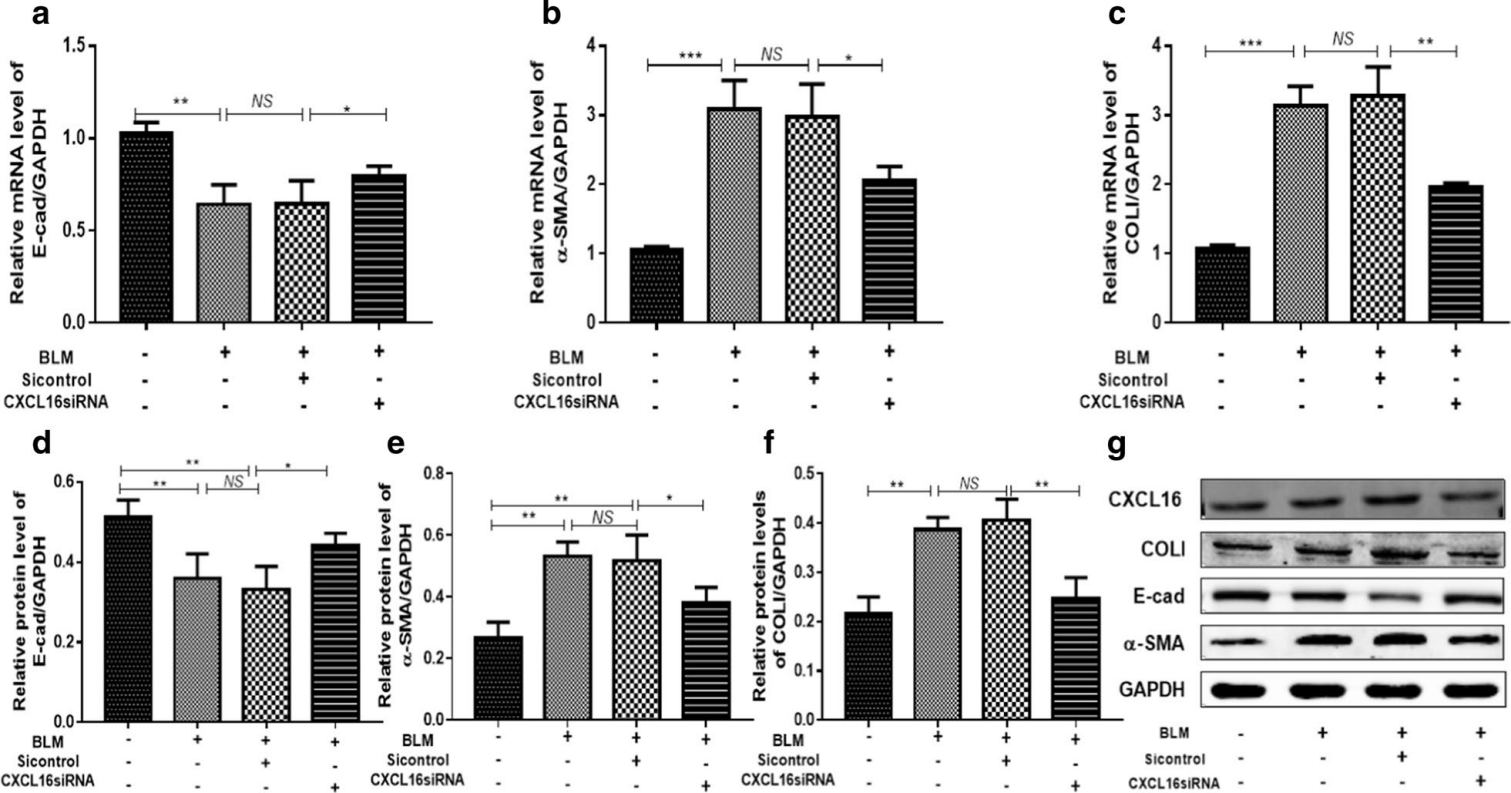
CXCL16 regulated BLM-induced EMT and ECM excretion in A549 cells. CXCL16 siRNA was transfected into A549 cells for 12 h, after treated the cells with 200 μg/ml BLM for 24 h. The mRNA levels of E-cad (**a**), α-SMA (**b**) and COLI (**c**) was determined by RT-PCR and the protein levels of E-cad (**d**), α-SMA (**e**) and COLI (**f**) was determined by western blot (**g**). *BLM* Bleomycin, *E-cad* E-cadherin, *α-SMA* α-smooth muscle actin, *COLI* collagen I; GAPDH served as a loading control. *P < 0.05, **P < 0.01, ***P < 0.001; *ns* not significant. Data represented three independent experiments

To investigate the specific role of CXCL16 in BLM-induced EMT, we silenced the expression of CXCL16 by transfecting A549 cells with CXCL16 siRNA, and the inhibitory efficiency of CXCL16 siRNA could be seen in Additional file 1: Fig. S2. RT-PCR, western blot and ELISA results showed that expression of CXCL16 was obviously down-regulated by CXCL16 siRNA in A549 cells. Expression of α-SMA and E-cadherin was measured in unstimulated and BLM stimulated A549 cells and the results showed that when CXCL16 was silenced, the expression of α-SMA was reduced (Fig. 4b, e), while the expression of E-cadherin was increased (Fig. 4a, d). Similarly, silencing CXCL16 with siRNA also reduced the expression of COLI induced by BLM, both in mRNA and protein levels (Fig. 4c, f). The above data indicated that CXCL16 might play an important role in modulating BLM-induced EMT and ECM excretion in human A549 cells.

### CXCL16 siRNA reduced production of pro-inflammatory and pro-fibrotic cytokines in human A549 cells

**Fig. 5:**
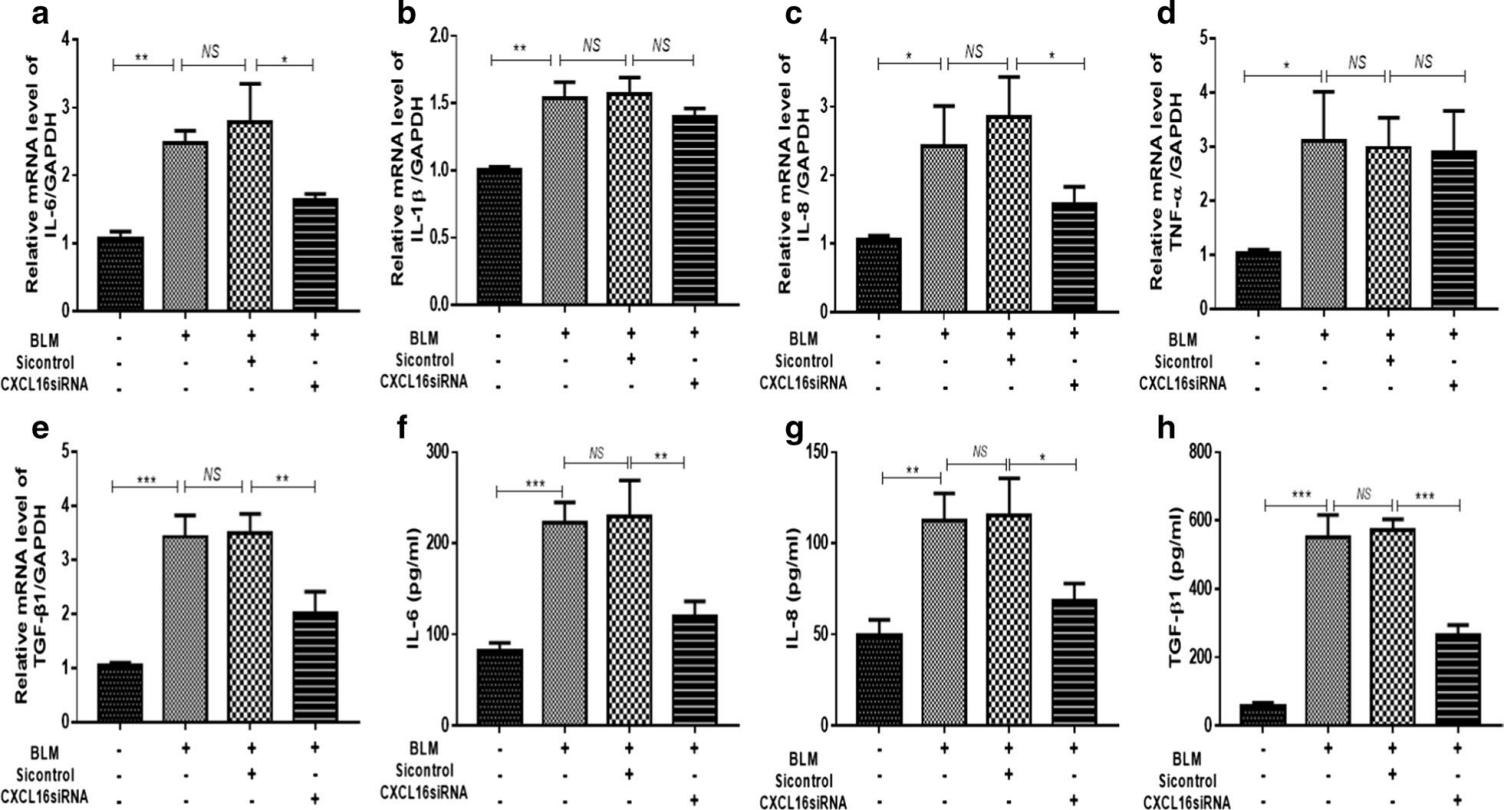
CXCL16 siRNA reduced production of pro-inflammatory and pro-fibrotic cytokines in A549 cells. After transfection with CXCL16siRNA for 12 h, A549 cells were stimulated with 200 μg/ml BLM for 24 h. The mRNA levels of IL-6 (**a**), IL-1β (**b**), IL-8 (**c**), TNF-α (**d**) and TGF-β1 (**e**) was determined by RT-PCR and the expression of IL-6 (**f**), IL-8 (**g**) and TGF-β1 (**h**) in the cell culture supernatant was detected by ELISA. *BLM* Bleomycin; Sicontrol: the control scramble siRNA; GAPDH served as the loading control. *P < 0.05, **P < 0.01, ***P < 0.001; *ns* not significant. Data represented three independent experiments

Pro-inflammatory and pro-fibrotic cytokines forms a complex regulation network during the occurrence and development of pulmonary fibrosis, whether CXCL16 modulate BLM-induced pulmonary fibrosis via promoting secretion of the pro-inflammatory and pro-fibrotic cytokines is still unknown. Expression of IL-6, IL-8, IL-1β, TNF-α and TGF-β1 was measured in unstimulated and BLM stimulated A549 cells and the results showed that after silencing of CXCL16, the expression of IL-6, IL-8 and TGF-β1 were markedly reduced in BLM stimulated A549 cells (Fig. 5a, c, e), while the expression of IL-1β and TNF-α had no obvious changes (Fig. 5b, d). Thus, we speculated that CXCL16 might modulate BLM-induced IL-6, IL-8 and TGF-β1 expression in A549 cells.

### CXCL16 may regulate EMT in human A549 cells via the TGF-β1/Smad3 pathways

**Fig. 6:**
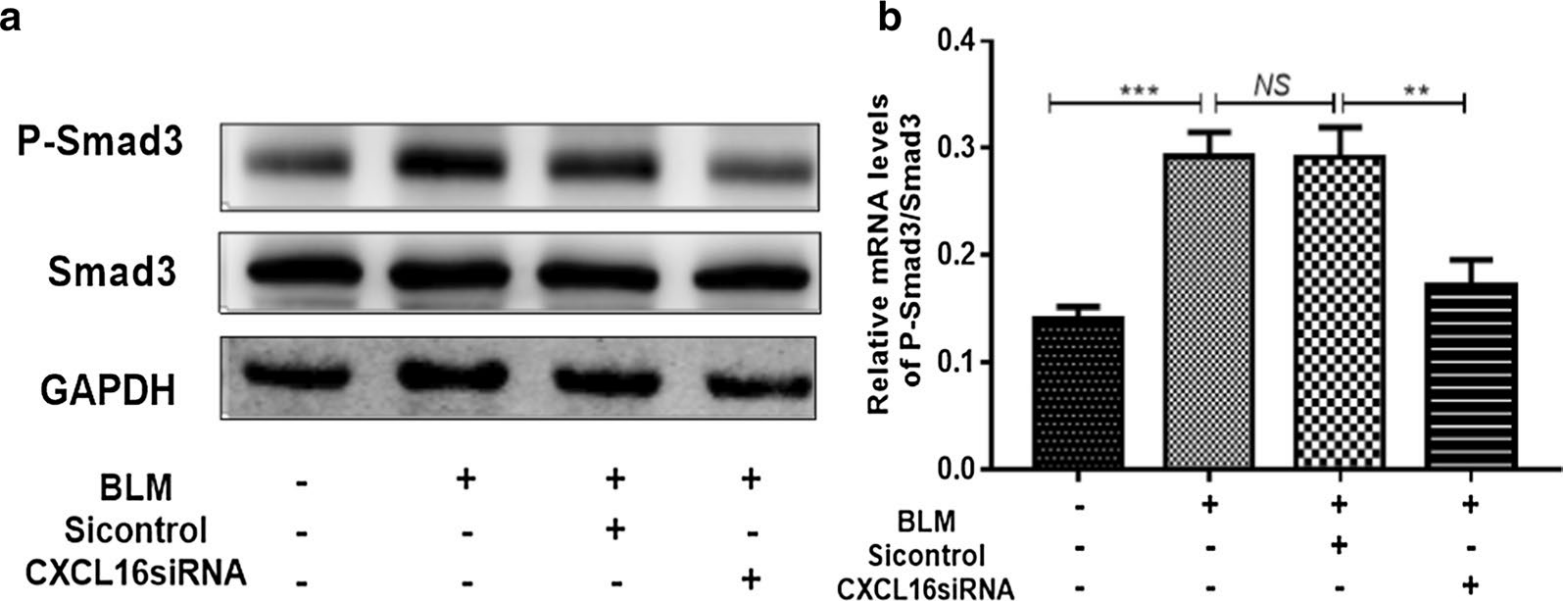
CXCL16 may regulate EMT in A549 cells via the TGF-β1/Smad3 pathways. P-Smad3 and Smad3 protein levels in A549 cells were detected by western blot after silencing of CXCL16. GAPDH served as a loading control. **P < 0.01, ***P < 0.001; *NS* not significant. Data represented three independent experiments

To further investigate the potential pathway by which CXCL16 regulates BLM-induced EMT, we detected the expression of TGF-β1 and Smad3 when CXCL16 was silenced in BLM-stimulated A549 cells. As shown in the results, the expression of TGF-β1 and the phosphorylation of Smad3 were markedly reduced when CXCL16 was silenced by siRNA in the A549 cells (Fig. 5e, h for TGF-β1; Fig. 6 for Smad3). These data indicated that CXCL16 might regulate the EMT process via the TGF-β1/Smad3 pathways.

## Discussion

Pulmonary fibrosis is the fundamental to the pathogenesis of many chronic pulmonary diseases, and always characterized by progressive decline in the lung functions. Although the molecular mechanism of pulmonary fibrosis remains unclear, the role of EMT in the pathogenesis of pulmonary fibrosis has become a new research hotspot [15, 16]. A recent study has demonstrated that the main features of fibrosis were the activation of fibroblast and the deposition of ECM, which occurs concomitant with proliferation and aggregation of MFB [17]. MFB acts as the main source of collagen in active pulmonary fibrosis and the main effector cells of pulmonary fibrosis [18]. Three sources of activated MFB have been found, including the proliferation and phenotypic transformation of fibroblasts around the injury site [19], the activation of EMT and the migration of fibrocytes [20, 21]. During the process of EMT, the down-regulation of epithelial markers (e.g., E-cadherin, ZO-1) and the up-regulation of mesenchymal markers (e.g., α-SMA, COLI) was observed, and epithelial cells loses polarity and recover its ability on migration, invasion and collagen production [22]. EMT is always involved in embryonic development, as well as in several pathological conditions, such as endometriosis, chronic obstructive pulmonary disease, metastasis of tumors and fibrosis in tissues or organs [15, 23–25]. It has been reported that CXCR6 could promote tumor metastasis through EMT [14]. CXCL16/CXCR6 participates in multiple organ fibrosis and plays a positive role in the occurrence of fibrotic diseases. But the previous studies of CXCL16/CXCR6 in fibrotic diseases mainly focused on the release of inflammatory factors, the aggregation and activation of immune cells and the migration of fibrocytes [26–28]. In our previous studies, sCXCL16 was overexpressed in serum of patients with rheumatoid arthritis-related interstitial lung disease, suggesting that it may play a role in the process of pulmonary fibrosis [29]. However, little is known whether CXCL16 is dysregulated in alveolar epithelial cells and the role of CXCL16 in modulating EMT in pulmonary fibrosis.

Repeated damage to the alveolar epithelial cells induced by various harmful factors has been accepted as the first and foremost cause of pulmonary fibrosis and complex networks of cytokines are involved in this process, as well as the subsequent tissue repair and fibrosis [30]. Here, a well-validated model of EMT in the A549 human alveolar cell line was used to study the mechanism of pulmonary fibrosis, for which a suite of morphological, phenotypic and functional markers and outcomes have been well characterized [31–33]. BLM is widely used as an inducer of pulmonary fibrosis in animal models [34], and similar effects were validated in vitro experiments, and EMT characteristics were detected in BLM treated A549 cells [35, 36]. Our research discovered that CXCL16 and its receptor CXCR6 were upregulated in BLM-induced EMT in the human A549 cells. We further verified the synergistic effect of CXCL16 and BLM in the process of pulmonary fibrosis.

To better understand the role of CXCL16 in BLM-induced EMT, we silenced the expression of CXCL16 by specific siRNA, then the occurrence of EMT and the production of collagens markedly reduced in BLM stimulated A549 cells. Our data indicated that CXCL16 might play an important role in modulating BLM-induced EMT and ECM excretion in A549 cells. Increasing evidence suggests that the important stimulator of collagen accumulation in pulmonary fibrosis is the increased production of pro-inflammatory and pro-fibrogenic cytokines/chemokines. The activation of MFB may be regulated by a complex network of various cytokines in local microenvironment. Some cytokines are involved in local injury and inflammation (e.g., IL-6, IL-1β, IL-8 and TNF-α), while others, such as TGF-β1, is mainly involved in tissue repair and fibrosis [37, 38]. Meanwhile, CXCL16 is closely related to the occurrence of inflammatory diseases and inflammatory-related tumors [39, 40], so here we detected the expression of above indicators in BLM-induced EMT in A549 cells. After silencing of CXCL16, the expression of IL-6, IL-8 and TGF-β1 were markedly reduced in BLM stimulated A549 cells. Thus, we speculated that CXCL16 might modulate BLM-induced IL-6, IL-8 and TGF-β1 expression in the A549 cells during EMT process.

Alveolar epithelial cells are one of the main cells that produce and secrete TGF-β1 in pulmonary fibrosis, and TGF-β1 is known to be the most critical fibrogenic factor, which could lead to the synthesis of collagen and other matrix proteins in large quantities. At present, the signal transduction pathways of TGF-β1 are mainly classified into Smad protein-dependent and -independent signaling and the TGF-β1-induced Smad3 signaling pathway has been considered to be one of the most important mechanisms of pulmonary fibrosis progression [41–43]. Further study of the regulatory mechanism of CXCL16 in BLM-induced EMT has been carried out and we found that the expression of TGF-β1 and the phosphorylation of Smad3 were significantly reduced in A549 cells when CXCL16 was silenced, which indicated that CXCL16 may regulate EMT in A549 cells via the TGF-β1/Smad3 signaling pathway.

There were also some limitations to the present study. First, A549 is a non-small-cell lung cancer cells, although it retains the characteristics of typical type II alveolar epithelial and acts as a well-studied model of EMT, it still behaves differently from normal epithelial cells in many occasions [44, 45]. Additional prospective studies with primary human lung alveolar type 2 cells are necessary to validate the role of CXCL16 in BLM-induced EMT. Second, we did not investigate the possible relationship between CXCL16 and other signal transduction pathways in EMT such as Wnt, Notch, Hedgehog, etc. Additional studies (e.g. in primary epithelial cells, animal models, human tissues) are necessary to elucidate the functional role of CXCL16 in modulating EMT in pulmonary fibrosis.

## Conclusions

In summary, we found that CXCL16 and its receptor CXCR6 were upregulated in BLM-induced EMT and synergistic effect of CXCL16 and BLM on the occurrence of EMT, the excretion of ECM, as well as the production of pro-inflammatory and pro-fibrotic cytokines in A549 cells have been observed. In addition, our study further elucidated that CXCL16 might regulate EMT in A549 cells via the TGF-β1/Smad3 pathways. Our results establish a new mechanism of BLM-induced EMT and may provide an important basis for the treatment of pulmonary fibrosis and related diseases. Further research is necessary to assess the role of CXCL16 in pulmonary fibrosis and its potential as a therapeutic target in fibrosis-related diseases.

## Supplementary Information

**Additional file 1:** **Table** **S1.** Information of the primer sequence. **Table** **S2.** Information of the antibodies. **Fig.** **S1.** CXCL16 and CXCR6 expression levels in the lung tissues of BLM-induced pulmonary fibrosis mice models. Representative serial lung sections from BLM-WT group and Saline-WT group were stained for CXCL16 and CXCR6. The results of immunohistochemistry showed that CXCL16/CXCR6 in the lung tissue of Salin-WT group was only expressed in a small amount in airway epithelial cells and vascular smooth muscle cells, while CXCL16/CXCR6 in the lung tissue of BLM-WT group was widely expressed in airway and alveoli epithelium cells and lung interstitium, accompanied by the infiltration of CXCR6 positive lymphocytes in the alveolar cavity. Saline-WT: Saline-treated wild-type mice; BLM-WT: Bleomycin-treated wild-type mice. N = 5, Scale bar = 100 μm. **Fig.** **S2.** Inhibitory efficiency of CXCL16 siRNA was observed by RT-PCR, western blot and ELISA 48 h after siRNA transfection. Sicontrol: the control scramble siRNA; Positive control: GAPDH siRNA; GAPDH served as a loading control. *P < 0.05, **P < 0.01, ***P < 0.001. ns, not significant. Data represented three independent experiments.

## Data Availability

The datasets used during the current study are available from the corresponding author on reasonable request.

## Abbreviations

EMT: Epithelial–mesenchymal transition
CXCL16: CXC chemokine ligand 16
CXCR6: C-X-C motif chemokine receptor 6
ECM: Extracellular matrix
BLM: Bleomycin
IL-6: Interleukin-6
IL-1β: Interleukin-1β
TNF-α: Tumor necrosis factor-alpha
TGF-β1: The transforming growth factor-β1
a-SMA: A-smooth muscle actin
COLI: Collagen I
