# Supplementary material for: Role of CXCL16 in BLM-induced epithelial–mesenchymal transition in human A549 cells
Source: Respir Res. 2021 Feb 6;22:42. doi: 10.1186/s12931-021-01646-7 (PMC7866482; doi:10.1186/s12931-021-01646-7)
Supplement: Supplementary file 1 — Additional file 1: Table S1. Information of the primer sequence. Table S2. Information of the antibodies. Fig. S1. CXCL16 and CXCR6 expression levels in the lung tissues of BLM-induced pulmonary fibrosis mice models. Representative serial lung sections from BLM-WT group and Saline-WT group were stained for CXCL16 and CXCR6. The results of immunohistochemistry showed that CXCL16/CXCR6 in the lung tissue of Salin-WT group was only expressed in a small amount in airway epithelial cells and vascular smooth muscle cells, while CXCL16/CXCR6 in the lung tissue of BLM-WT group was widely expressed in airway and alveoli epithelium cells and lung interstitium, accompanied by the infiltration of CXCR6 positive lymphocytes in the alveolar cavity. Saline-WT: Saline-treated wild-type mice; BLM-WT: Bleomycin-treated wild-type mice. N = 5, Scale bar = 100 μm. Fig. S2. Inhibitory efficiency of CXCL16 siRNA was observed by RT-PCR, western blot and ELISA 48 h after siRNA transfection. Sicontrol: the control scramble siRNA; Positive control: GAPDH siRNA; GAPDH served as a loading control. *P < 0.05, **P < 0.01, ***P < 0.001. ns, not significant. Data represented three independent experiments. [file 12931_2021_1646_MOESM1_ESM.docx]

Additional file:

Table S1. Information of the primer sequence

| Gene names |  | Primer sequence |
| --- | --- | --- |
| CXCL16 | forward primer  reverse primer | 5'-CCCGCCATCGGTTCAGTTC-3'  5'-CCCCGAGTAAGCATGTCCAC-3' |
| CXCR6 | forward primer  reverse primer | 5'-CAAAGCATCTCTGCTGGTGTTC-3'  5'-TACATGCAGGGCAGAAAGACC-3' |
| α-SMA | forward primer  reverse primer | 5'-CTATGAGGGCTATGCCTTGCC-3'  5'-GCTCAGCAGTAGTAACGAAGGA-3' |
| TGF-β1 | forward primer  reverse primer | 5'-TGGTGGAAACCCACAACGAA-3'  5'-GAGCAACACGGGTTCAGGTA-3' |
| Collagen I | forward primer  reverse primer | 5'-GATGGACTCAACGGTCTCCC-3'  5'-CTTCTCTTGAGGTGGCTGGG-3' |
| IL-6 | forward primer  reverse primer | 5’-AACCTGAACCTTCCAAAGATGG-3’ 5’-TCTGGCTTGTTCCTCACTACT-3’ |
| IL-8 | forward primer  reverse primer | 5’-CATACTCCAAACCTTTCCACCCC-3’  5’-TCAGCCCTCTTCAAAAACTTCTCCA-3’ |
| TNF-α | forward primer  reverse primer | 5’-TGGCGTGGAGCTGAGAGATA-3’  5’-TGATGGCAGAGAGGAGGTTG-3’ |
| IL-1β | forward primer  reverse primer | 5’-TGAGCTCGCCAGTGAAATGA-3’  5’-AGATTCGTAGCTGGATGCCG-3’ |
| E-cadherin | forward primer  reverse primer | 5'- AGGCTAGAGGGTCACCGCGTC-3'  5'- GCTTTGCAGTTCCGACGCCAC-3' |
| GAPDH | forward primer  reverse primer | 5'-CCCGCCATCGGTTCAGTTC-3'  5'-CCCGCCATCGGTTCAGTTC-3' |

Table S2. Information of the antibodies

| Name of products | Catalogue numbers | Manufacturers |
| --- | --- | --- |
| Anti-α-Smooth Muscle Actin antibody | 19245S | Cell Signaling Technology (USA) |
| Anti-Phospho-Smad3 antibody | 9520 | Cell Signaling Technology (USA) |
| Anti-Smad3 antibody | 9513 | Cell Signaling Technology (USA) |
| Anti-E-cadherin antibody | [20874-1-AP](http://www.ptgcn.com/products/E-cadherin-Antibody-20874-1-AP.htm) | Proteintech (USA) |
| Anti-Collagen I antibody | [14695-1-AP](http://www.ptgcn.com/products/COL1A2-Antibody-14695-1-AP.htm) | Proteintech (USA) |
| Anti-CXCL16 antibody | ab101404 | Abcam (USA) |
| Anti-CXCR6 antibody | ab8023 | Abcam ( USA) |
| Anti-GAPDH antibody | C1312 | Solarbio (China) |


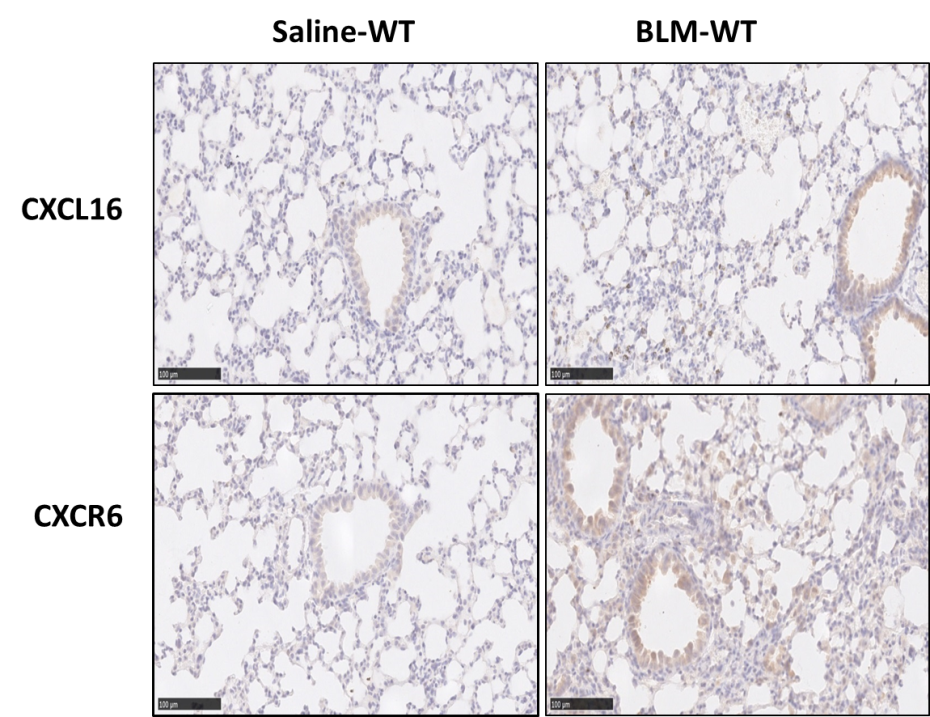


Fig.S1. CXCL16 and CXCR6 expression levels in the lung tissues of BLM-induced pulmonary fibrosis mice models. Representative serial lung sections from BLM-WT group and Saline-WT group were stained for CXCL16 and CXCR6. The results of immunohistochemistry showed that CXCL16/CXCR6 in the lung tissue of Salin-WT group was only expressed in a small amount in airway epithelial cells and vascular smooth muscle cells, while CXCL16/CXCR6 in the lung tissue of BLM-WT group was widely expressed in airway and alveoli epithelium cells and lung interstitium, accompanied by the infiltration of CXCR6 positive lymphocytes in the alveolar cavity. Saline-WT: Saline-treated wild-type mice; BLM-WT: Bleomycin-treated wild-type mice. N=5, Scale bar=100μm.


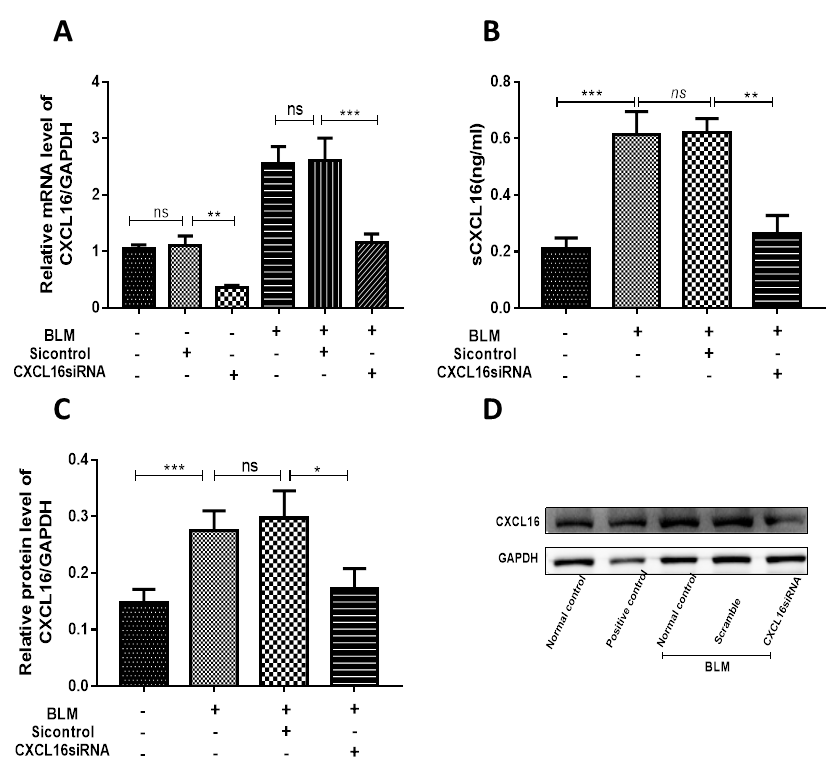


Fig.S2. Inhibitory efficiency of CXCL16 siRNA was observed by RT-PCR, western blot and ELISA 48 h after siRNA transfection. Sicontrol: the control scramble siRNA; Positive control: GAPDH siRNA; GAPDH served as a loading control. *P< 0.05, **P< 0.01, ***P< 0.001. ns, not significant. Data represented three independent experiments.
